# Time-resolved synchrotron X-ray total scattering of alkali-activated materials with high and low calcium contents

**DOI:** 10.1107/S1600577526006776

**Published:** 2026-08-03

**Authors:** Hyeonseok Jee, Nishant Garg

**Affiliations:** a Department of Civil and Environmental Engineering University of Illinois Urbana-Champaign Urbana IL61801 USA; University of Malaga, Spain

**Keywords:** pair distribution function, alkali-activated materials, reaction kinetics, isothermal calorimetry

## Abstract

The reaction kinetics and structure of alkali-activated binders are known to be affected by their calcium (Ca) contents. However, the intrinsic difference between high Ca and low Ca alkali-activated systems, especially at the atomic scale, has not been fully investigated. Here, we compare local atomic ordering and reaction kinetics of blast furnace slag and metakaolin as representatives of high and low Ca systems, respectively. *In situ*X-ray pair distribution function (PDF) analysis and isothermal calorimetry were utilized in parallel to investigate the evolution of local structure in these systems. Isothermal calorimetry results showed contrasting reaction kinetics of high Ca (alkali-activated slag) and low Ca (alkali-activated metakaolin) systems, where the latter shows no induction period. The evolution of specific PDF peaks is related to the main binder gels in each respective system and can be used to track their reaction kinetics, exhibiting excellent correlation with the cumulative heat data (*R*^2^ > 0.97). Finally, and most importantly, the PDF results revealed that the atomic ordering of N-A-S-(H) gel is limited up to ∼10 Å, whereas C-(N)-A-S-H gel grew up to ∼40 Å, which suggests that there is an intrinsic difference in the formation mechanism and local structures of dominant gels formed in high and low Ca systems.

## Abstract 2

Synchrotron *in situ* high-energy X-ray total scattering/pair distribution function (PDF) analysis quantifies the time-resolved structural evolution of high and low calcium alkali-activated binders, with PDF peak evolution tracking reaction progress and correlating with calorimetry (*R*^2^ > 0.97). The measurements resolve distinct medium-range ordering in the primary gels, demonstrating the sensitivity of synchrotron PDF for probing disordered cementitious phases.

## 1. Introduction

Global demand for physical infrastructure is robust and expected to grow considerably, given the world population is expected to increase by 1.7 billion by the year 2050 (United Nations, 2022 ▸). Ordinary Portland cement (OPC) continues to be the most widely used binder for concrete, mortar, blocks, and plaster applications (Habert *et al.*, 2020 ▸). Specifically, since the last decade, nearly four billion tons of OPC have continued to be produced annually worldwide, and its dominance is expected to continue due to its good mechanical performance and economic feasibility (Miller *et al.*, 2021 ▸). Despite several benefits of OPC, its production process, unfortunately, accounts for up to 6–8% of anthropogenic carbon dioxide emissions (Schneider, 2019 ▸). This has accelerated interest in alternative binders such as alkali-activated materials and geopolymers, which can provide comparable mechanical performance with reduced CO_2_ emissions (Provis, 2018 ▸). However, most of the alkali-activated binders derive their strength from poorly crystalline or X-ray amorphous reaction products, which are difficult to investigate using conventional diffraction methods.

In this context, total scattering combined with atomic pair distribution function (PDF) analysis has become a powerful method for characterizing disordered and nanocrystalline materials, including cementitious binders (du Plessis *et al.*, 2011 ▸; Cuesta *et al.*, 2019 ▸; Kwon *et al.*, 2019 ▸). Unlike conventional Bragg diffraction, which primarily probes long-range periodic order, total scattering captures both Bragg and diffuse scattering. Fourier transformation of the total scattering signal yields the PDF, *G*(*r*), which describes the probability of finding atom pairs separated by a real-space distance *r*. Consequently, PDF analysis provides direct insight into local coordination environments (short-range order) and structural connectivity over nanometre length scales (medium-range order), which are critical for the binding gels that govern the performance of alkali-activated systems. Synchrotron radiation is particularly advantageous to PDF measurements since high-energy X-rays enable access to large momentum transfer *Q*, improving real-space resolution, and high flux supports rapid acquisition for time-resolved, *in situ* studies of hydration and hardening.

PDF methods have been applied widely to materials in which functional properties are governed by local structure rather than long-range crystallinity, including batteries (Christensen *et al.*, 2019 ▸), xerogel (Petkov *et al.*, 2002 ▸), nanomaterials (Jensen *et al.*, 2016 ▸), and metal–organic frameworks (Firth *et al.*, 2021 ▸). In cement and concrete research, synchrotron- and neutron-based PDF analyses are increasingly used to characterize amorphous and nanocrystalline reaction products and to resolve dynamic processes during setting and hardening. Prior studies have shown that *in situ* PDF can track the evolution of atomic correlations during cement hydration and related phenomena, including carbonation (Morandeau & White, 2015 ▸), retardation (Garg & White, 2017 ▸), shrinkage (Yang *et al.*, 2018 ▸), mechanical deformation (Bae *et al.*, 2018 ▸; Jee *et al.*, 2020 ▸), and overall reaction kinetics for a range of systems (White *et al.*, 2011 ▸, 2013 ▸; Peys *et al.*, 2019 ▸; Gong & White, 2022 ▸). Collectively, these studies demonstrate that PDF enables continuous quantification of structural evolution even when the dominant products lack long-range order.

Alkali-activated binders are well suited to PDF analysis since their reactants and primary binding phases are typically low-crystallinity gels. Alkali activation involves mixing an alkaline activator (often NaOH and/or sodium silicate) with a reactive aluminosilicate precursor, driving dissolution followed by precipitation and condensation reactions that form a hardened binder (Provis & Bernal, 2014 ▸). Precursor chemistry strongly influences the resulting gel products, with Ca content serving as a key variable. High Ca precursors (*e.g.* slag) commonly form alkali-containing calcium aluminosilicate hydrate [C-(N)-A-S-H]-type gels with local environments resembling poorly crystalline calcium silicate hydrate (C-S-H)-like structures (Richardson *et al.*, 1994 ▸; Wang *et al.*, 1995 ▸; Fernández-Jiménez *et al.*, 2003 ▸; Myers *et al.*, 2013 ▸), whereas low Ca precursors (*e.g.* metakaolin) typically yield highly disordered alkaline aluminosilicate [N-A-S-(H)] gels (Duxson *et al.*, 2007 ▸; Khale & Chaudhary, 2007 ▸; Provis & Bernal, 2014 ▸). However, the extent of short- and medium-range ordering in these gels, and its evolution over time, needs to be further understood in the atomic scale. Moreover, increasing Ca content may induce transitions in binding environments and lead to contrasting durability properties (Bernal *et al.*, 2013 ▸; Sankar *et al.*, 2018 ▸) highlighting the need for methods that can quantify atomic-scale structure continuously during reaction.

**Figure 1:**
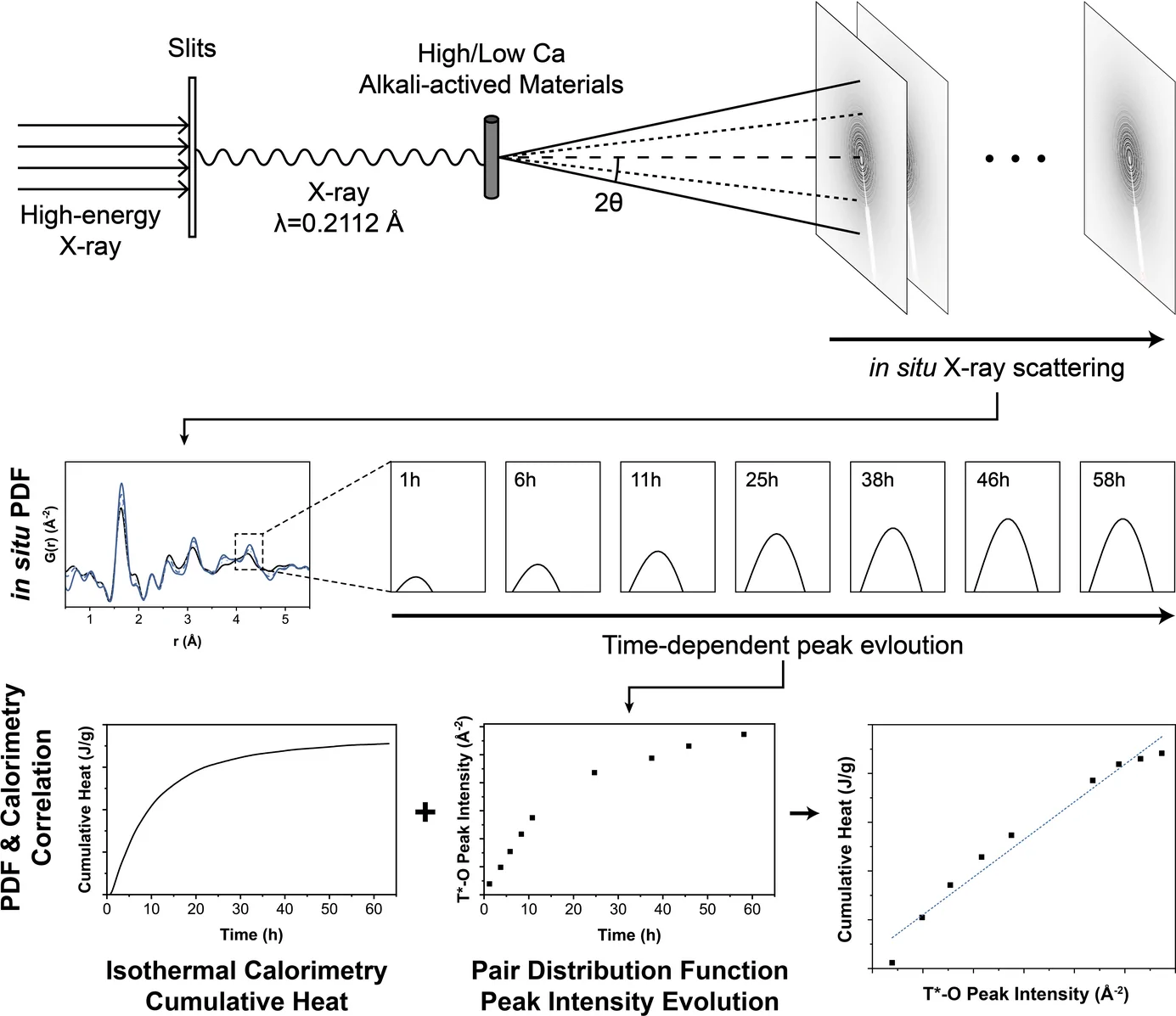
Schematic of the synchrotron *in situ* high-energy X-ray total scattering and pair distribution function (PDF) workflow used to track reaction kinetics in alkali-activated binders with contrasting calcium contents. The total scattering data are Fourier transformed to obtain the PDF from which the time-dependent evolution of selected PDF peaks (associated with the formation of the primary binder gels) is quantified. PDF-derived peak intensity evolution is compared with isothermal calorimetry cumulative heat release, enabling a direct correlation between structural development measured by PDF and macroscopic reaction progress.

While previous PDF studies have provided important insight into the reaction progress and local structural evolution of individual alkali-activated systems, a direct time-resolved comparison of high Ca and low Ca systems under the same PDF–calorimetry framework remains limited. In particular, the spatial extent of gel-associated atomic correlations of the main binder gels has not been systematically compared during reaction. Here, we employ synchrotron-based *in situ*X-ray total scattering and PDF analysis to compare reaction kinetics and atomic ordering in representative high and low Ca alkali-activated binders, as shown in Fig. 1 ▸. Slag and metakaolin were selected as model precursors for the high Ca and low Ca systems, respectively, and the time evolution of PDF peak intensities was analyzed alongside isothermal calorimetry to relate heat-release signatures to atomistic structural development. Calorimetric response and PDF peak evolution correlate strongly (*R*^2^ = 0.97–0.99 for both systems), demonstrating that PDF provides a quantitative, time-resolved structural measure of reaction progress. The results reveal distinct early age kinetics, including a pronounced induction period in the high Ca system (up to ∼8 h) that is not observed in the low Ca system under the conditions studied. PDF analysis further resolves contrasting medium-range order in the primary binding gels: atomic correlations in the C-(N)-A-S-H gel extend to ∼40 Å in the high Ca system, whereas correlations in the N-A-S-(H) gel are limited to ∼10 Å even after extended curing (five months). Collectively, these findings demonstrate the ability of synchrotron PDF to quantify both kinetics and nanoscale structural development in alkali-activated binders and to elucidate how Ca content governs gel ordering and reaction pathways.

## 2. Materials and methods

### 2.1. Materials and sample preparation

Two types of experimentally synthesized alkali-activated materials, which represent high and low Ca systems, were prepared for this study: alkali-activated slag (AAS) and alkali-activated metakaolin (AAM). The slag precursor was activated with the sodium silicate solution to formulate AAS. To make a sodium silicate solution, sodium metasilicate (Na_2_SiO_3_, CAS No. 6834–92–0, Sigma-Aldrich) was dissolved in deionized water (18 MΩ cm resistivity) and homogenized for 24 h using a magnetic stirrer bar. The ratio of Na_2_O and slag was 4% (4 g of Na_2_O per 100 g of slag), and the water-to-binder ratio (w/b) was maintained at 0.44. The oxide composition of slag used in this study was as follows: 43.0 wt% CaO, 32.6 wt% SiO_2_, 10.3 wt% MgO, 7.5 wt% Al_2_O_3_, 1.2 wt% SO_3_ and 5.4 wt% other elements. MetaMax from BASF was used as metakaolin (2SiO_2_·Al_2_O_3_) in this study. Sodium silicate solution was used as an alkaline activator for metakaolin. To prepare the sodium silicate solution, sodium hydroxide pellets (CAS No. 1310-73-2, Sigma-Aldrich) were first dissolved in water, and sodium silicate liquid (‘D’ product, PQ Corporation) was added into the solution and mixed with a magnetic stirrer bar for 24 h. The chemical composition of the sodium silicate liquid was 29.4 wt% SiO_2_, 14.7 wt% Na_2_O, and 55.9 wt% H_2_O with a SiO_2_/Na_2_O weight ratio of 2. The activating solution for metakaolin had a Na_2_O/SiO_2_ molar ratio of 0.5, and the w/b ratio was 0.9. The Na_2_O/metakaolin ratio was maintained at 0.28 (28 g of Na_2_O per 100 g of metakaolin).

### 2.2. High-energy X-ray total scattering and pair distribution function analysis

For *in situ* X-ray scattering experiments, slag and metakaolin were mixed with an alkaline activator in 50 ml polypropyl­ene centrifuge tubes. The tubes were homogenized with a vortex mixer for one minute at 2500 rpm, taking one minute of rest, followed by another minute of mixing at 2500 rpm. Prepared AAS and AAM pastes were then loaded into a polyimide capillary (Masterflex Transfer Tubing, Cole-Parmer, USA) with a 1 mm diameter via suction. Both ends of the polyimide capillary were immediately sealed with quick-setting ep­oxy.

High-energy *in situ*X-ray total scattering experiments were conducted on the 11-ID-B beamline at the Advanced Photon Source, Argonne National Laboratory, at the high energy of 58 keV (λ = 0.2112 Å). The X-ray scattering signals were collected with an amorphous silicon imaging plate X-ray detector (XRD1621, Perkin-Elmer, USA) (Chupas *et al.*, 2003 ▸). The distance from the test samples to the imaging plate detector was set to 180 mm. X-ray scattering data were collected for a duration of 2 min at selected times, and *in situ*X-ray data were acquired up to 81.5 h and 58.1 h for AAS and AAM, respectively. The *Fit2D* program (Hammersley, 2016 ▸, 1997 ▸) was used for the conversion of 2D to 1D data, and the data was calibrated with CeO_2_. Background signals were corrected by subtracting the scattering contribution from an empty polyimide capillary measured under the same experimental geometry.

Atomic PDF [*G*(*r*)] data were obtained from the X-ray scattering results with the *PDFgetX2* software (Qiu *et al.*, 2004 ▸). Equation (1) shows the Fourier transform of the experimentally measured total scattering structure factor, *S*(*Q*), with the momentum-transfer vector (*Q*). The maximum *Q* cutoff for the conversion of X-ray scattering data was 24 Å^−1^. The instrumental parameters were refined with a CeO_2_ standard (Sigma-Aldrich) using the *PDFgui* software (Farrow *et al.*, 2007 ▸). Peak positions in the *G*(*r*) curves were determined by searching for the maximum *G*(*r*) value within a predefined *r* range for each atom–atom correlation,
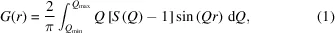
where *G*(*r*) = PDF results, and *Q* = 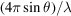.

### 2.3. Isothermal calorimetry

Heat evolution of AAS and AAM was assessed with an isothermal calorimeter (TAM Air, TA Instruments, USA). To conduct the isothermal calorimetry measurement, 5 g of each AAS and AAM paste were prepared for the test. Initial heat flows of AAM and AAS were measured for ∼85 h at 23°C. Since the AAM and AAS samples were mixed with an alkaline solution outside of the calorimeter, and inserting the samples in the calorimeter can significantly disrupt initial heat flow, the first 1 h data was omitted. The cumulative heat of the reaction was calculated based on the obtained heat flow.

## 3. Results and discussion

### 3.1. Reaction kinetics of alkali-activated slag and metakaolin via isothermal calorimetry

Here, we plan to investigate the difference between the heat curves of a high Ca and a low Ca system.

**Figure 2:**
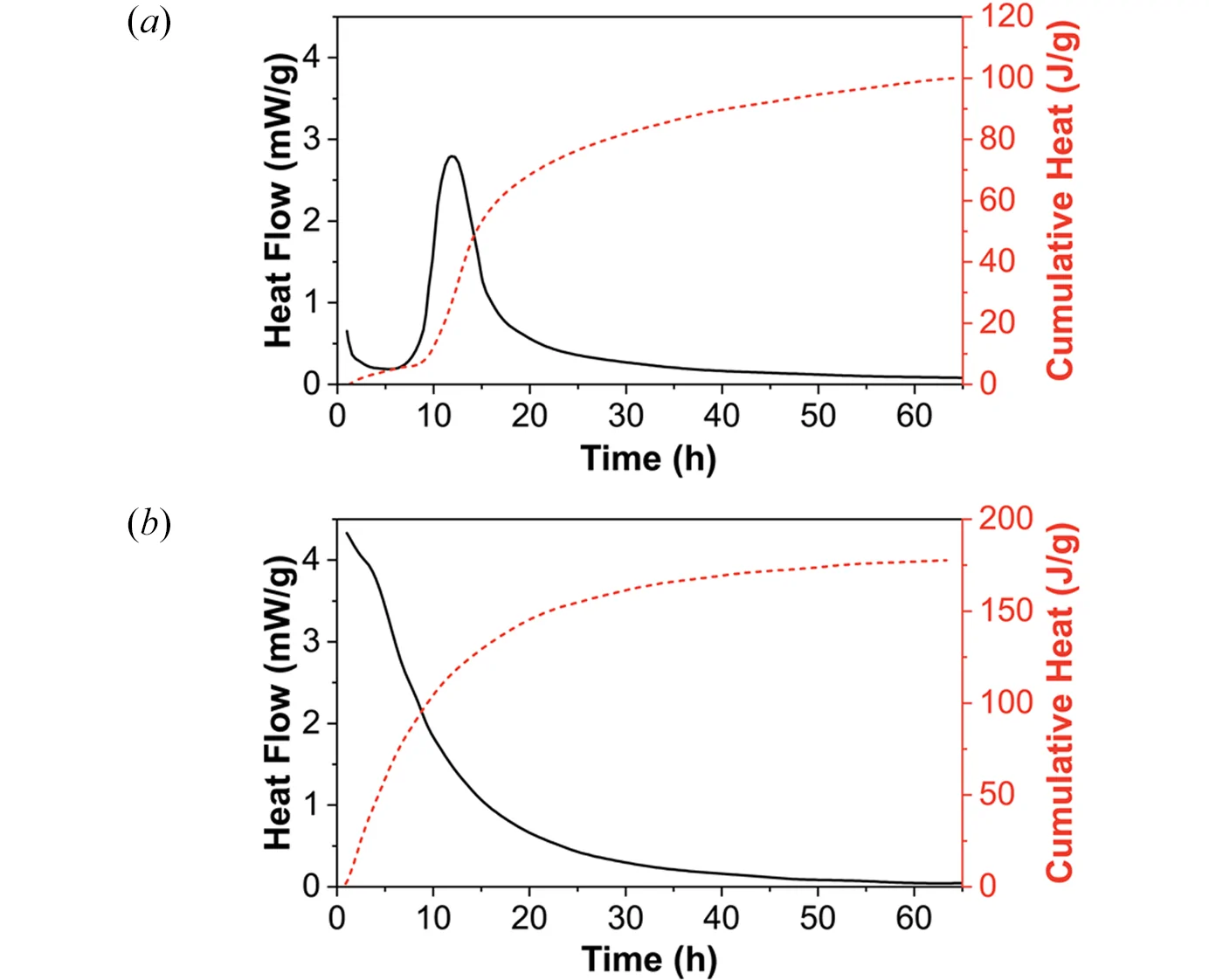
Measured heat-flow and cumulative heat curves for (*a*) AAS (high Ca) and (*b*) AAM (low Ca).

Fig. 2 ▸ depicts the heat flow curves and cumulative heat of the AAM and AAS examined with isothermal calorimetry. As shown in the figure, the type of solid precursors significantly affects the early heat evolution behavior. The initial heat evolution of AAS had a distinct second peak with the induction period (∼7 h), which is similar to the hydration behavior of ordinary Portland cement. On the other hand, the heat evolution of AAM showed a single exothermic peak without the induction period. Accordingly, the cumulative heat curves of AAS and AAM [Fig. 2 ▸(*b*)] showed contrasting behaviors. While the cumulative heat curve of AAS showed an S-shaped curve due to the presence of an induction period, the cumulative heat curve of AAM had a logarithmic type curve. Sun and Vollpracht observed strong dissolution period peaks before 1 h of reaction in the NaOH-activated metakaolin and ground-granulated blast furnace slag (Sun & Vollpracht, 2018 ▸). These exothermic peaks are related to the initial dissolution of the aluminosilicate precursors in the alkali-activated solutions. However, these peaks were not observed in our work due to the omission of the initial 1 h data.

Several studies have pointed out the different reaction behaviors of alkali-activated systems with regard to the Ca content in the systems. Sankar *et al.* (2018 ▸) investigated the early reaction kinetics of alkali-activated slag-fly ash systems. They revealed the formation of the C-N-S-H soft gel when the Ca contents were in the systems, whereas such a gel was not observed in the low Ca systems. Moreover, another study showed that the presence of Ca ions in the solid precursors served a crucial role in the retarding effect of zinc oxide on alkali-activated materials (Garg & White, 2017 ▸). The role of Ca in controlling early age reaction pathways has also been reported in several alkali-activated systems. Lee & van Deventer (2002 ▸) suggested that Ca-containing salts can shorten setting time by providing heterogeneous nucleation centers in alkali-activated fly ash systems. Suraneni *et al.* (2014 ▸) also showed that Ca(OH)_2_ substitution substantially accelerated the setting of metakaolin geopolymer and made its stiffening behavior similar to that of slag geopolymer. More recently, Chen *et al.* (2018 ▸) reported that calcium addition increased the rate and extent of metakaolin dissolution, thereby modifying the solution chemistry and accelerating geopolymer gel formation. These studies suggest that calcium can affect reaction kinetics not only by changing the final gel chemistry but also by modifying precursor dissolution, nucleation, and gel polymerization. Similarly, in this study, the early reaction behaviors of alkali-activated materials highly depend on the presence of calcium ions in solid precursors. The contrasting kinetics of AAS and AAM can therefore be interpreted as reflecting different gel-forming pathways: nucleation and growth of C-(N)-A-S-H-type products in the high Ca system, and dissolution and repolymerization of aluminosilicate species into an N-A-S-(H) framework in the low Ca system. The early reaction behaviors of the two systems will be further scrutinized on an atomistic scale via total X-ray scattering and PDF analysis in the following sections.

### 3.2. X-ray scattering and pair distribution function

The total X-ray scattering method and PDF analysis have been important tools to examine the crystalline structure and atomic scale ordering of materials (Warren *et al.*, 1936 ▸). Since PDF analysis can resolve the local atomic environment of non-crystalline structures, it has been an invaluable technique for the alkali-activated system in which precursors and final gel products are generally amorphous or nano-crystalline. Furthermore, the rapid data acquisition with a synchrotron X-ray source allowed researchers to analyze the real-time reaction progress of alkali-activated systems (Garg & White, 2017 ▸; Gong & White, 2022 ▸). Here, the crystalline structure and local atomic structure development of AAS and AAM paste were investigated using *in situ*X-ray scattering and PDF analysis.

**Figure 3:**
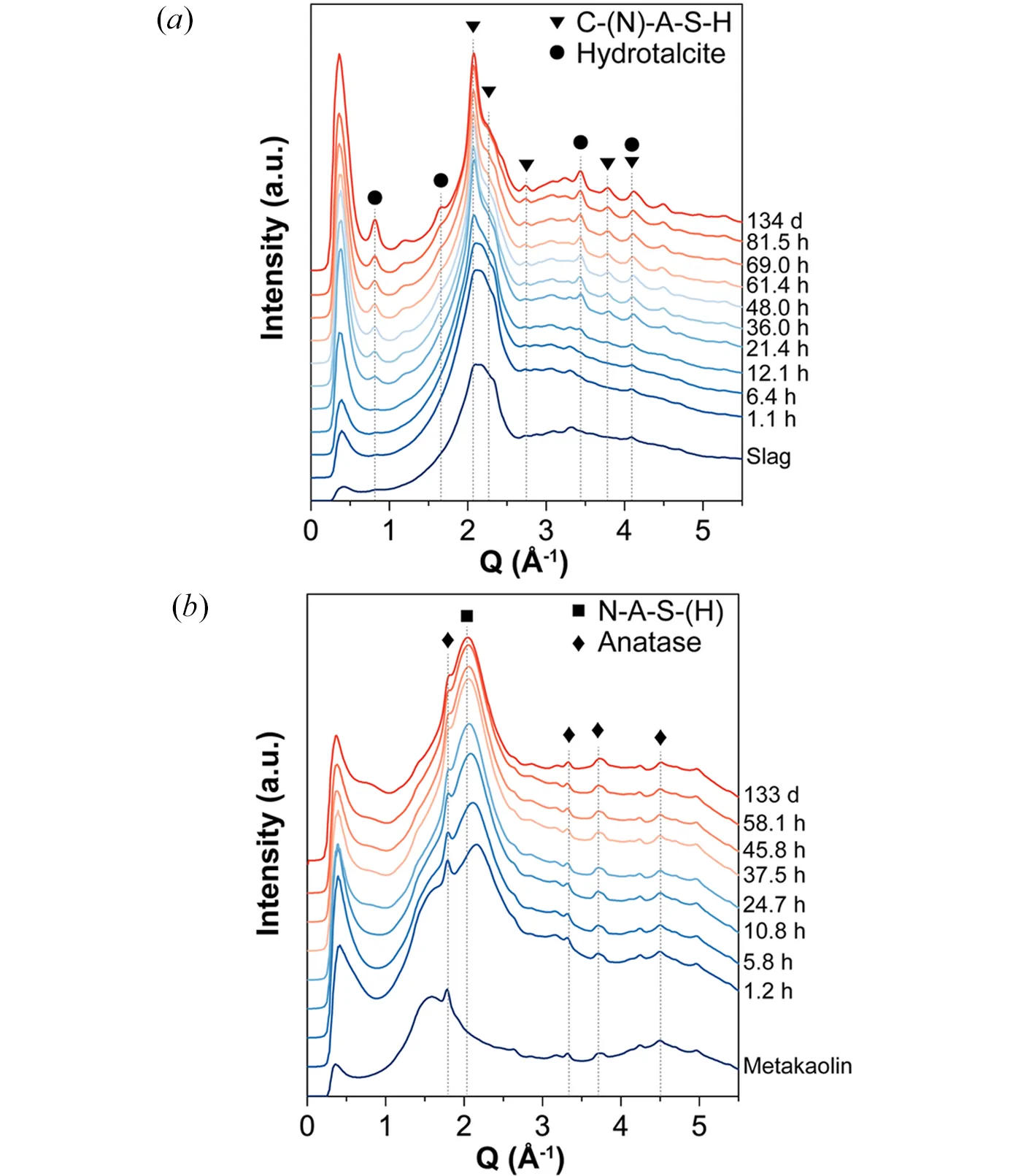
High-energy X-ray scattering patterns of (*a*) AAS (high Ca) and (*b*) AAM (low Ca). In the AAS, C-(N)-A-S-H gel and hydro­talcite (ICSD No. 107626; Taylor, 1973 ▸) were identified. In the AAM, N-A-S-(H) gel and anatase (ICSD No. 63711; Howard *et al.*, 1991 ▸) phase were found.

Fig. 3 ▸(*a*) depicts the *in situ*X-ray scattering patterns of the AAS at the selected reaction time. In Fig. 3 ▸(*a*), the X-ray scattering curve of the precursor of the AAS showed a broad diffuse peak centered at ∼2.2 Å^−1^, indicating that the unreacted slag lacked crystallinity. The unreacted slag peak intensities decreased with the progress of the alkali-activated reaction. Concurrently, the X-ray scattering peaks of C-(N)-A-S-H gel (1.20, 2.05, 2.30, 2.65, 3.70, 4.15 and 4.50 Å^−1^) (Geng *et al.*, 2017 ▸; Myers *et al.*, 2015*a* ▸) and hydro­talcite (Ht, Mg_6_Al_2_(OH)_16_CO_3_·4.5H_2_O, ICSD No. 107626) phase (0.78, 1.20, 1.68, 3.05, 3.48 and 4.15 Å^−1^) (Taylor, 1973 ▸) were identified as the reaction progressed. C-(N)-A-S-H gel is the main binder gel in alkali-activated slag and is generally described as a poorly crystalline, layered C-S-H/tobermorite-like structure in which Al and alkali cations can be incorporated into C-S-H frameworks (Richardson *et al.*, 1994 ▸; Fernández–Jiménez *et al.*, 2003 ▸; Myers *et al.*, 2013 ▸). The peak intensities of reaction products [C-(N)-A-S-H gel and Ht] constantly evolved with the reaction up to ∼134 days. Moreover, the low-*Q* region (*Q* < ∼0.6 Å^−1^) showed an increase in intensity as the reaction progressed. This low-*Q* region includes the 002 basal reflection of C-S-H-type gel (*Q* = 0.45–0.50 Å^−1^), corresponding to a basal spacing of approximately 12–14 Å (Taylor, 1997 ▸; Grangeon *et al.*, 2013 ▸). This basal spacing reflects the tobermorite-like layered structure of C-S-H-type gels and can vary with gel chemistry and interlayer structure, including Ca/Si ratio, Al/Si ratio, alkali content, interlayer water content, and the presence of cross-linking (Richardson, 2014 ▸; Myers *et al.*, 2015*a* ▸; Myers *et al.*, 2015*b* ▸; Garg *et al.*, 2019 ▸). Thus, the increase in low-*Q* intensity is consistent with the formation and increasing medium-range ordering of C-(N)-A-S-H gel, although the exact peak position could not be analyzed because this region was partially obscured by the strong intensity near the direct beam.

The X-ray scattering patterns of the AAM paste with the progress of the reaction are plotted in Fig. 3 ▸(*b*). The metakaolin precursor used in this study showed a broad hump centered at ∼1.6 Å^−1^. Some trace peaks are identified as characteristic peaks of TiO_2_ (anatase, ICSD No. 63711; Howard *et al.*, 1991 ▸), which was presented in the metakaolin precursor as an impurity. Since anatase is chemically inert, the peaks from TiO_2_ did not change during the reaction, indicating the TiO_2_ phase was not involved in the alkali-activated reaction. Except for the peaks originating from anatase, the lack of crystalline peaks in the precursor suggests that the structure of the metakaolin is largely amorphous. After the addition of sodium hydroxide solution for the alkali activation, another broad diffuse peak located at ∼2.05 Å^−1^ emerged. This is a typical diffraction pattern of the reaction product of the low calcium alkali-activated precursor, namely N-A-S-(H) gel (Abdelrahman & Garg, 2022 ▸). N-A-S-(H) gel is known as the main binder phase of alkali-activated metakaolin, whose local atomic structure corresponds to a disordered zeolite structure (Provis *et al.*, 2005 ▸). The gradual increase of N-A-S-(H) peak height and sharpness indicated the continued formation of N-A-S-(H) gel during the reaction, together with the gradual reduction of the metakaolin-derived diffuse hump. (Yang *et al.*, 2000 ▸; Zhan *et al.*, 2002 ▸).

**Figure 4:**
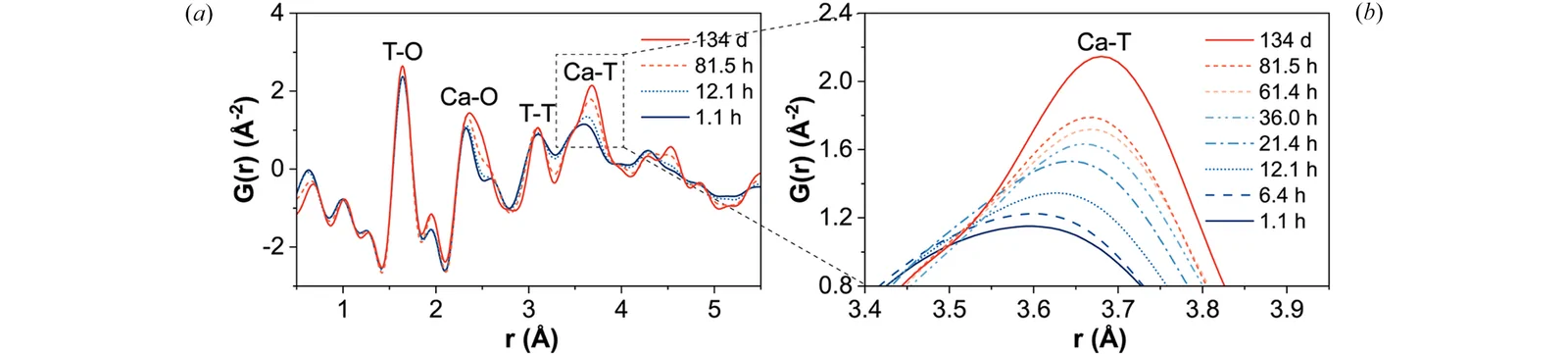
*In situ*X-ray pair distribution function of AAS. (*a*) Local atomic correlations over 0.5 to 5.5 Å. (*b*) The evolution of the Ca–T peak.

The X-ray scattering data were Fourier transformed to obtain the pair distribution function curves at selected times and reported in Figs. 4 ▸ and 5 ▸. The corresponding longer *r*-range PDFs of AAS and AAM pastes are provided in Fig. S1 to show the persistence of atomic correlations beyond the local structure region. The longer-range ordering behavior of the primary reaction gels is discussed in more detail in Section 3.4. All PDFs were generated using a *Q*_max_ value of 24 Å^−1^. Fig. 4 ▸ displays the *in situ*X-ray PDF results of AAS at the selected time in the short *r* range (*r* < 5.5 Å). The local atomic structure of the AAS paste showed the atom–atom correlation of T–O, Ca–O, T–T, and Ca–T peaks, where the symbol T indicates tetrahedral atoms such as silicon and aluminium, which were assigned based on previous literature (Garg & White, 2017 ▸; Grangeon *et al.*, 2017 ▸; Gong & White, 2018 ▸; McCaslin *et al.*, 2025 ▸). Recently, Gong and White have shed light on the PDF peak assignments of NaOH-activated slag by simulating the molecular structure of slag (Gong & White, 2022 ▸), which enabled more accurate atom–atom correlation assignments. The peaks located below the first T–O peak (∼1.6 Å) originate from the truncation errors during the PDF analysis and convey no structural meaning (Egami & Billinge, 2012 ▸). On the other hand, the peak assignments for the atom–atom correlation peaks above 5.5 Å were challenging due to the significant overlap between several atoms.

The local atomic structure of the AAS paste clearly showed the evolution of reaction products during the activation of slag. The intensity of the Ca–O peak (∼2.4 Å) significantly increased with the alkali-activated reaction, which is consistent with previous PDF results on alkali-activated slag systems (Garg & White, 2017 ▸; Gong & White, 2022 ▸). The evolution of the nearest Ca-O correlation intensity is likely to be due to (i) an increase in the coordination of calcium atoms and/or (ii) a more ordered calcium environment during the alkali activation. More importantly, the Ca–T peak located at ∼3.7 Å appeared to drastically increase over time, as shown in Fig. 4 ▸(*b*). The Ca–T peak in the alkali-activated slag has been assigned to the nearest calcium and tetrahedral Si and Al correlation in C-(N)-A-S-H gel (Garg & White, 2017 ▸; Gong & White, 2016 ▸). Apart from the primary Ca–T correlation, the recent PDF study on sodium hydroxide-activated slag revealed that the atom–atom correlations of the second nearest T–O and the nearest Ca–Ca also contribute to the PDF peak located at ∼3.7 Å (Gong & White, 2022 ▸), which is also attributed to the newly formed C-(N)-A-S-H gel structure. Thus, the growth of a peak at ∼3.7 Å can be a direct indication of the formation of nano-crystalline C-(N)-A-S-H gel in AAS systems.

**Figure 5:**
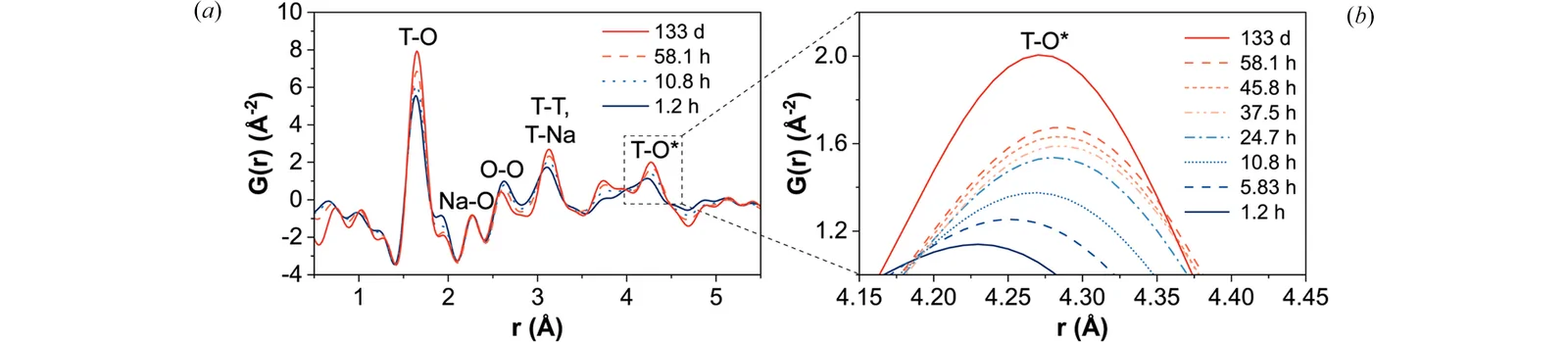
*In situ*X-ray pair distribution functions of AAM. (*a*) Local atomic correlations over 0.5 to 5.5 Å. (*b*) The evolution of the T–O* peak.

Fig. 5 ▸(*a*) shows the *in situ*X-ray PDF curves for the local atomic structure evolution of AAM paste up to 133 days. The overall trends of the atomic structure evolution of AAM were similar to the previous PDF studies (White *et al.*, 2011 ▸; White *et al.*, 2013 ▸; Garg & White, 2017 ▸). The PDF peaks of AAM were assigned based on the previous studies on alkali-activated metakaolin (Garg & White, 2017 ▸; White *et al.*, 2013 ▸). Although a small quantity of anatase was identified in the metakaolin precursor via X-ray diffraction, the atom–atom correlations from anatase did not clearly appear in the PDF results, possibly due to its small abundance. X-ray diffraction analysis also revealed that the anatase phase was not significantly involved in the alkali-activation reaction. Hence, the effect of anatase was disregarded in the following PDF analysis.

The peak located at ∼1.6 Å represents the nearest correlation of oxygen and tetrahedral atoms (silicon and aluminium). The Si–O contribution is typically located near ∼1.60 Å, whereas tetrahedral Al^IV^–O correlations in aluminosilicate frameworks occur at slightly longer distances, around ∼1.7–1.8 Å (Baur & Fischer, 2019 ▸). Higher-coordinated Al–O environments in metakaolin appear at longer distances near ∼1.9 Å (White *et al.*, 2010*a* ▸). In the present data, a distinct Al^IV^–O shoulder could not be clearly resolved from the main T–O peak, likely due to overlap with the Si–O contribution, structural disorder, and the finite real-space resolution associated with *Q*_max_ = 24 Å^−1^. Unlike the AAS paste, which showed no significant change in the intensity of the nearest T–O peak, the peak intensity of the T–O peak increased over time in the AAM system. One of the possible explanations for the growth of the T–O peak in the AAM system is the change of aluminium coordination during the formation of the N-A-S-(H) gel structure. Common metakaolin has a 2D sheet structure with tetrahedral, pentahedral, and octahedral aluminium coordination (White *et al.*, 2010*a* ▸; White *et al.*, 2010*b* ▸). During alkali activation, the pentahedrally and octahedrally coordinated aluminium dissolves from the metakaolin structure and forms a three-dimensional N-A-S-(H) gel structure (Provis & Van Deventer, 2009 ▸). Moreover, Garg & Skibsted (2019 ▸) revealed that the pentahedrally coordinated aluminium site is significantly responsible for the reactivity and dissolution of metakaolin. Hence, the growth of the first T–O peak height is attributed to the dissolution of Al^V^ and Al^VI^ species in metakaolin and the formation of Al^IV^ in the newly formed aluminosilicate framework. The decrease of Al–O peak intensity located at ∼1.9 Å, which originated from the higher coordinated aluminium in the metakaolin structure, also supports the dissolution and repolymerization of aluminium species. Lastly, it has been recently shown that metakaolin layers undergo a nano-scale thinning process during the dissolution phase, which may also contribute to this change in the T–O peak observed here (Romero & Garg, 2022 ▸).

In the higher-*r* region, the feature near ∼2.3 Å is assigned to Na–O correlations, while the broad feature near ∼2.7 Å is mainly associated with O–O correlations. The feature near ∼3.1 Å is primarily assigned to T–T correlations, with possible contribution from T–Na correlations associated with charge-balancing Na in the aluminosilicate framework. These assignments are based on previous *in situ* neutron and X-ray PDF studies of metakaolin-based geopolymers and partial PDF simulations of metakaolin and hydro­sodalite-like aluminosilicate framework structures (White *et al.*, 2011 ▸; White *et al.*, 2013 ▸; Garg & White, 2017 ▸). As the reaction progressed, the atom–atom correlation near ∼2.7 Å, which is primarily associated with O–O correlations in the aluminosilicate framework, showed a gradual decrease as the peak shifted to the lower-*r* region. Previous partial PDF analysis on alkali-activated metakaolin revealed that the Al–Al atom correlation also contributed to this *r* region, which originated from the geometrically strained aluminium in the metakaolin structure (White *et al.*, 2013 ▸). Therefore, the decrease and shift of this feature could be interpreted as reflecting the dissolution and reorganization of strained Al environments in the metakaolin precursor, superimposed on the O–O correlations of the evolving aluminosilicate framework. Concurrently, the peak located at ∼3.1 Å showed a notable increase, which has the atom–atom correlation of T–T and Na–T. The growth of this peak height indicates the formation of aluminosilicate networks as well as the inclusion of Na ions as the charge compensator in the geopolymer framework structure as the reaction progresses.

Lastly, the PDF peak at ∼4.3 Å has been mainly assigned to the T–O* correlation associated with the aluminosilicate framework of the N-A-S-(H) gel, which is plotted in Fig. 5 ▸(*b*). The asterisk sign was added to differentiate this peak from the first T–O correlation at ∼1.6 Å. The T–O* peak is associated with locally ordered four-membered aluminosilicate rings in the N-A-S-(H) structure (White *et al.*, 2013 ▸). The T–T distance at 4.42 Å across the diagonal of four-membered rings in a hydro­sodalite-like structure can also contribute to this *r*-region (Murshed & Gesing, 2008 ▸). Hence, the growth of the T–O* correlation also indicates the evolution of the N-A-S-(H) aluminosilicate structure. Previous PDF studies also suggested that the T–O* correlation in the 4.15–4.35 Å region can directly relate to the reaction kinetics of alkali-activated metakaolin (White *et al.*, 2011 ▸; Garg & White, 2017 ▸), which will be further discussed in the following section.

### 3.3. Correlation between PDF and isothermal calorimetry

Previous *in situ* PDF analysis studies on alkali-activated slag (Garg & White, 2017 ▸; Gong & White, 2022 ▸) and metakaolin (Garg & White, 2017 ▸; White *et al.*, 2011 ▸; White *et al.*, 2013 ▸) suggested that the development of specific peak intensities can reflect the reaction kinetics of those materials. In this section, we attempt to correlate the cumulative heat data from isothermal calorimetry and the atomic structure development obtained via PDF analysis.

**Figure 6:**
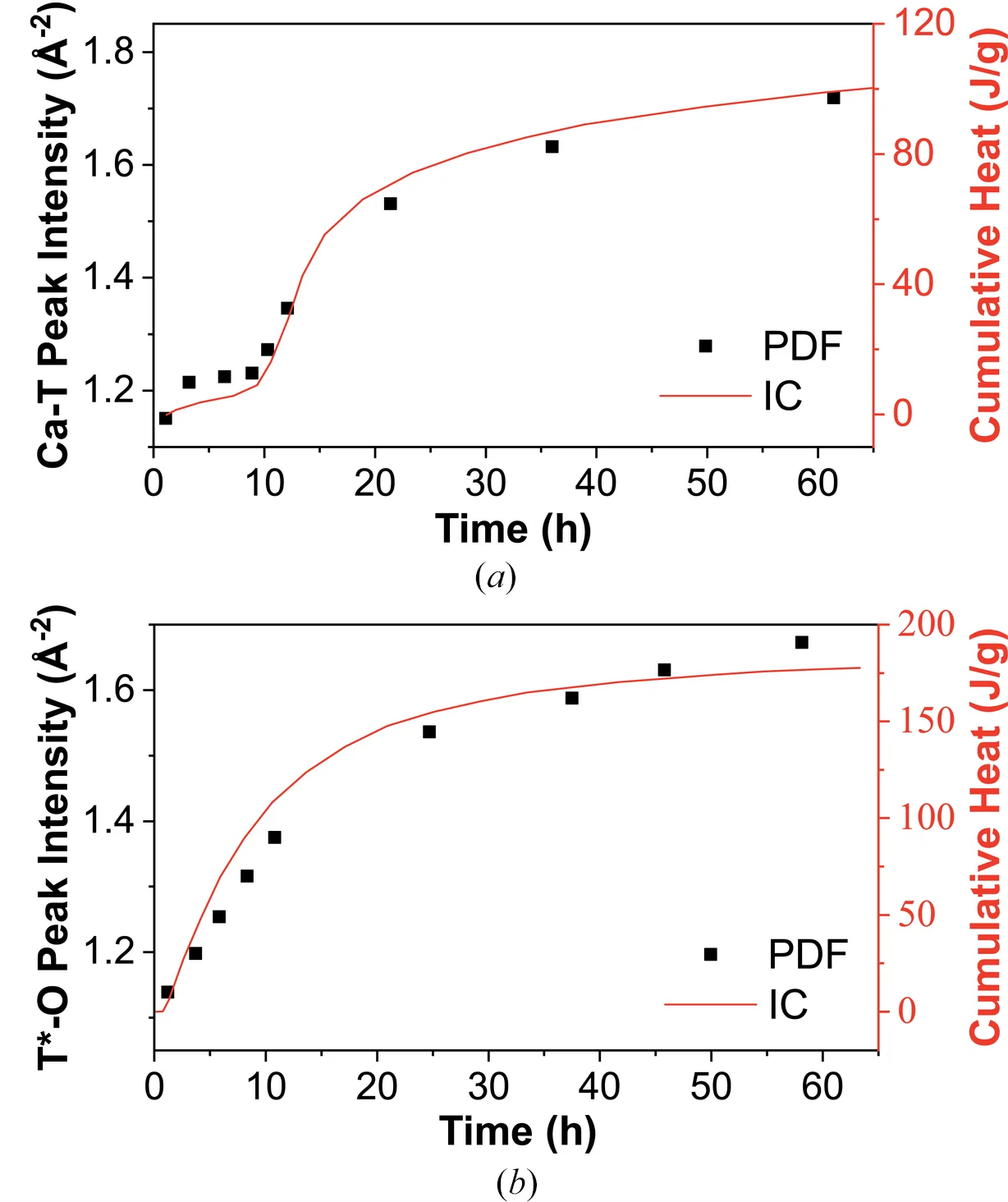
(*a*) Cumulative heat data and the intensity of the Ca–T peak of AAS. (*b*) Cumulative heat data and the intensity of the T–O* peak of AAM. Cumulative heat data was obtained via isothermal calorimetry (IC), and peak intensities were obtained via pair distribution function (PDF) analysis.

Fig. 6 ▸(*a*) depicts the cumulative heat evolution of AAS obtained by isothermal calorimetry and the evolution of the Ca–T peak in PDF results located at ∼3.7 Å, respectively. It can be clearly observed that there is a strong correlation between the reaction kinetics obtained from two different techniques. As discussed in Section 3.2, the PDF peak intensities of Ca–T correlation in the alkali-activated slag system reflect the formation and growth of the C-(N)-A-S-H gel, which is the primary reaction product of the slag system. Thus, the heat evolution of the AAS system has a substantial relationship with the formation of the primary gel.

Similar to the AAS system, a strong correlation between the main product and the cumulative heat in the AAM system was observed. Fig. 6 ▸(*b*) shows the cumulative heat from isothermal calorimetry and the peak intensities evolution of the T–O* peak (∼4.3 Å) from PDF results in the AAM system, respectively. As shown in the figure, the evolution of the T–O* peak, which is the signature peak of the main reaction gel in the AAM system, was directly correlated with the cumulative heat obtained from isothermal calorimetry data. This result indicates that the main exothermic process in alkali-activated metakaolin is mainly attributed to the geopolymerization process and the formation of the main reaction gel product.

**Figure 7:**
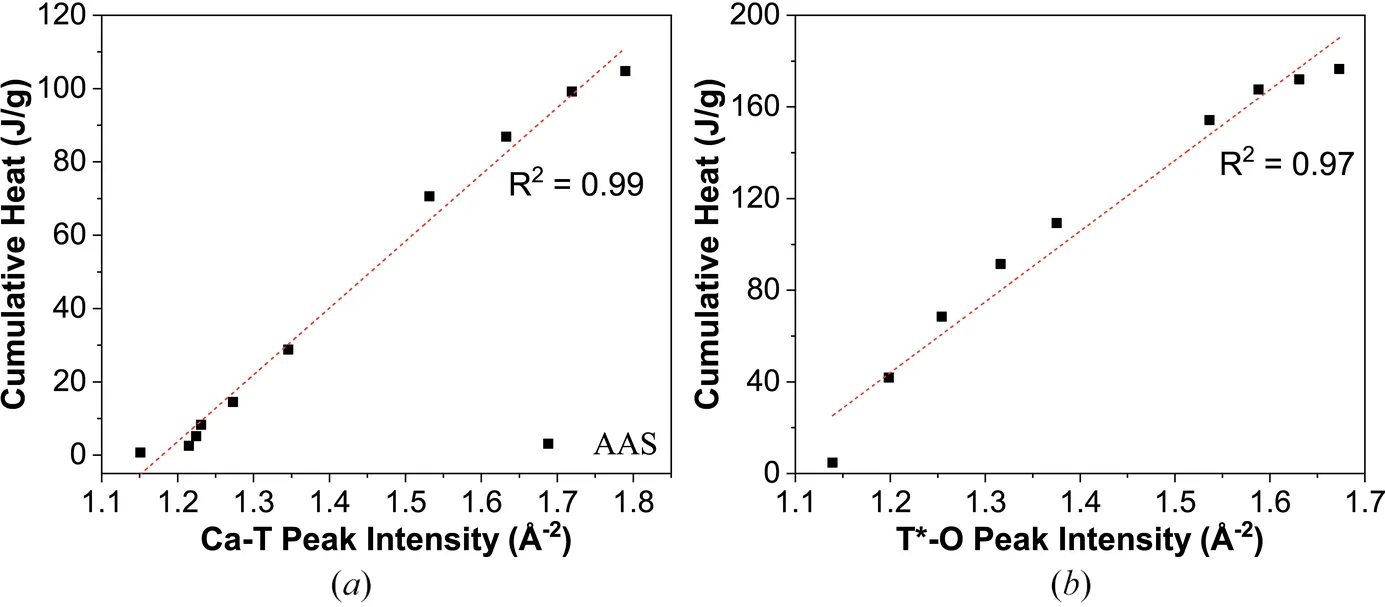
Comparison between the cumulative heat from isothermal calorimetry data and (*a*) Ca–T correlation peak intensities in AAS paste and (*b*) T–O* correlation peak intensities in AAM paste obtained by PDF analysis.

In Fig. 7 ▸, the comparison between cumulative heat data and the intensity of atom–atom correlations from the PDF results is plotted. Fig. 7 ▸(*a*) compares the Ca–T peak intensity with cumulative heat for AAS, whereas Fig. 7 ▸(*b*) compares the T–O* peak intensity with cumulative heat for AAM. As shown in the figure, the close alignment between isothermal calorimetry data and PDF peak intensities in AAM and AAS systems was further clearly observed. For both AAM and AAS systems, there were strong linear correlations between PDF and cumulative heat with high *R*^2^ values. Although linear fitting showed good correlations between two different techniques for both alkali-activated systems, it was found that the logarithmic shape functions can better fit the correlation between PDF and calorimetry results in AAM systems. Similarly, the correlation between the two techniques in AAS systems can be more accurately captured by sigmoidal functions. In this study, we could not figure out the underlying mechanisms of these trends; thus, future research will be needed to further elucidate these gaps.

The possibilities of using PDF peak intensities as the indicators of reaction degrees in the alkali-activated system have been reported in our previous study (Garg & White, 2017 ▸). In this study, the usage of Ca–T and T–O* correlation was validated for tracking the C-(N)-A-S-H and N-A-S-(H) gel formation and reaction progress in the alkali-activated system with a more comprehensive data set. By combining atomic-scale simulation and *in situ*X-ray PDF analysis, a recent study (Gong & White, 2022 ▸) has also successfully investigated the degree of reaction of the NaOH-activated slag system. The degree of reaction of slag obtained by PDF analysis showed a strong correlation with other characterization techniques, such as isothermal calorimetry and Fourier transform infrared spectroscopy. Furthermore, the peak intensities of specific PDF atom–atom correlations related to the formation of C-(N)-A-S-H gel showed good agreement with cumulative heat from isothermal calorimetry data. The present results build on these previous studies by directly comparing representative high Ca and low Ca alkali-activated systems under the same PDF–calorimetry framework. In both systems, the time evolution of gel-associated PDF correlations closely follows the cumulative heat release, indicating that the main heat-evolving process is strongly linked to the formation of the dominant binder gel. This comparison highlights that PDF peak evolution can serve not only as a structural descriptor, but also as a time-resolved indicator of reaction progress across alkali-activated systems with different gel chemistries.

### 3.4. Nano-structural evolution of slag and metakaolin

In Section 3.3, it has been suggested that the reaction kinetics of the alkali-activated materials system are significantly related to the evolution of atom–atom correlations of the main reaction products in PDF results. These main reaction products include C-(N)-A-S-H gel and N-A-S-(H) gel for AAS (high Ca) and AAM (low Ca) systems, respectively. To further investigate the atomic-scale structural evolution of these gels during the *in situ* reaction, we attempted to isolate the PDF curves of the gel products by subtracting the PDF curves of unreacted precursor phases as well as crystalline products.

In the X-ray scattering curves of AAS paste [Fig. 3 ▸(*a*)], three different phases were identified: unreacted slag precursor, C-(N)-A-S-H gel, and hydro­talcite. To isolate the PDF pattern of C-(N)-A-S-H gel, the PDF curves originating from the unreacted slag and hydro­talcite phases were subtracted from the *in situ* PDF data. The PDF patterns of the hydro­talcite phase were calculated by refining the PDF data attributed to hydro­talcite (ICSD No. 107626; Taylor, 1973 ▸) via *PDFgui* software (Farrow *et al.*, 2007 ▸). The PDF refinement for hydro­talcite was conducted for the *in situ* PDF data in the 40–60 Å range. The unreacted slag was largely amorphous, and the atom–atom correlations of the slag precursor in this study were present up to ∼20 Å. The C-(N)-A-S-H gel formed in the high Ca alkali-activated materials systems is a C-S-H (I)-type gel with sodium and aluminium ions substitution (Skibsted & Andersen, 2013 ▸; Hong & Glasser, 2002 ▸; Puertas *et al.*, 2011 ▸). The PDF of synthetic C-S-H (I) has shown that the structure of C-S-H (I) was present up to ∼35 Å, and the structural correlations diminished above that region (Skinner *et al.*, 2010 ▸). Moreover, it has been revealed that alkali ions can decrease the atomic-scale structural ordering of C-(N)-A-S-H gel (Garg *et al.*, 2019 ▸). Hence, unreacted slag and C-(N)-A-S-H gel would have limited contribution to the atom–atom correlations past ∼40 Å, and the 40–60 Å range in PDF patterns of AAS paste will be mostly attributed to the hydro­talcite phase. Unlike the hydro­talcite phase, the direct simulation of unreacted slag and evolved C-(N)-A-S-H gel was not possible in this study due to the lack of exact structural information on those phases. Gong & White (2016 ▸) introduced the indirect method for quantifying the unreacted slag content in the alkali-activated slag system. Similarly, in this study, the content of unreacted slag in the AAS pastes with the function of time was estimated by fitting the atom–atom correlation peaks between the range of 2.8–3.8 Å. The PDF peak centered at ∼3.42 Å, which is tentatively assigned as Mg-Si/Al correlation in the slag, was used for estimating the content of the unreacted precursor.

Similar to the AAS system, the structural details of the unreacted precursor and the main binder gel of the AAM system are not explicitly disclosed. Therefore, a similar methodology with AAS paste was applied to investigate the quantification of unreacted metakaolin precursor in the AAM paste. The PDF peak fitting was conducted for the range of 1.4–2.1 Å with the Gaussian function. The atom–atom correlation located at ∼1.92 Å was utilized to estimate the unreacted metakaolin content, which represents the Al–O correlation in the metakaolin precursor (White *et al.*, 2011 ▸; White *et al.*, 2013 ▸; White *et al.*, 2010*a* ▸; White *et al.*, 2010*b* ▸). It should be noted that this subtraction procedure was used to obtain residual PDF signals associated with the reaction gels, rather than to perform a full quantitative phase refinement. Because the unreacted precursors and reaction gels are poorly crystalline and have overlapping atom-pair correlations, the estimated precursor contributions should be considered semi-quantitative. Therefore, the residual PDF signals are used here primarily to compare the relative extent of gel-associated atomic ordering, not to determine absolute phase fractions or unique gel structures.

**Figure 8:**
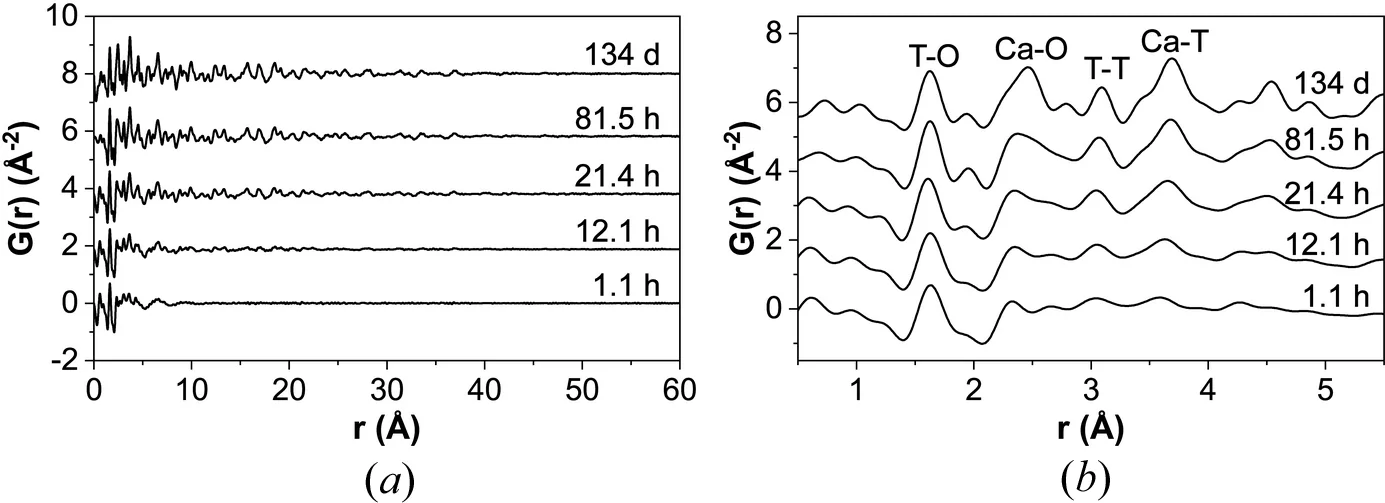
The pair distribution function curves of C-(N)-A-S-H gel in the AAS paste over (*a*) long *r* range (0–60 Å) and (*b*) short *r* range (0.5–5.5 Å). The curves were obtained by subtracting the unreacted slag and secondary crystalline phase (hydro­talcite) from the original pair distribution patterns at the specific reaction time.

Figs. 8 ▸ and 9 ▸ plot the evolution of the main binder gels in AAS and AAM pastes, respectively, by subtracting the unreacted precursors and secondary phase. In the long range [Figs. 8 ▸(*a*) and 9 ▸(*a*)], a significant difference between the nano-scale ordering of two alkali-activated systems was observed. In the AAS paste, the atomic ordering of C-(N)-A-S-H gel remains in the short-range as the reaction commences (∼1.1 h), and PDF patterns show no structural correlation above ∼10 Å. As the reaction progresses, the atomic correlations of C-(N)-A-S-H gradually extend to longer *r* ranges, which had atomic ordering up to ∼40 Å at 134 days of reaction. In contrast, N-A-S-(H) gel in the AAM paste shows no drastic change in the long *r*-range structure. At 133 days of reaction, the atomic ordering of N-A-S-(H) gel showed only short-range correlations up to ∼10 Å, suggesting that N-A-S-(H) gel in the AAM paste remains largely amorphous even after long-term reaction progression.

**Figure 10:**
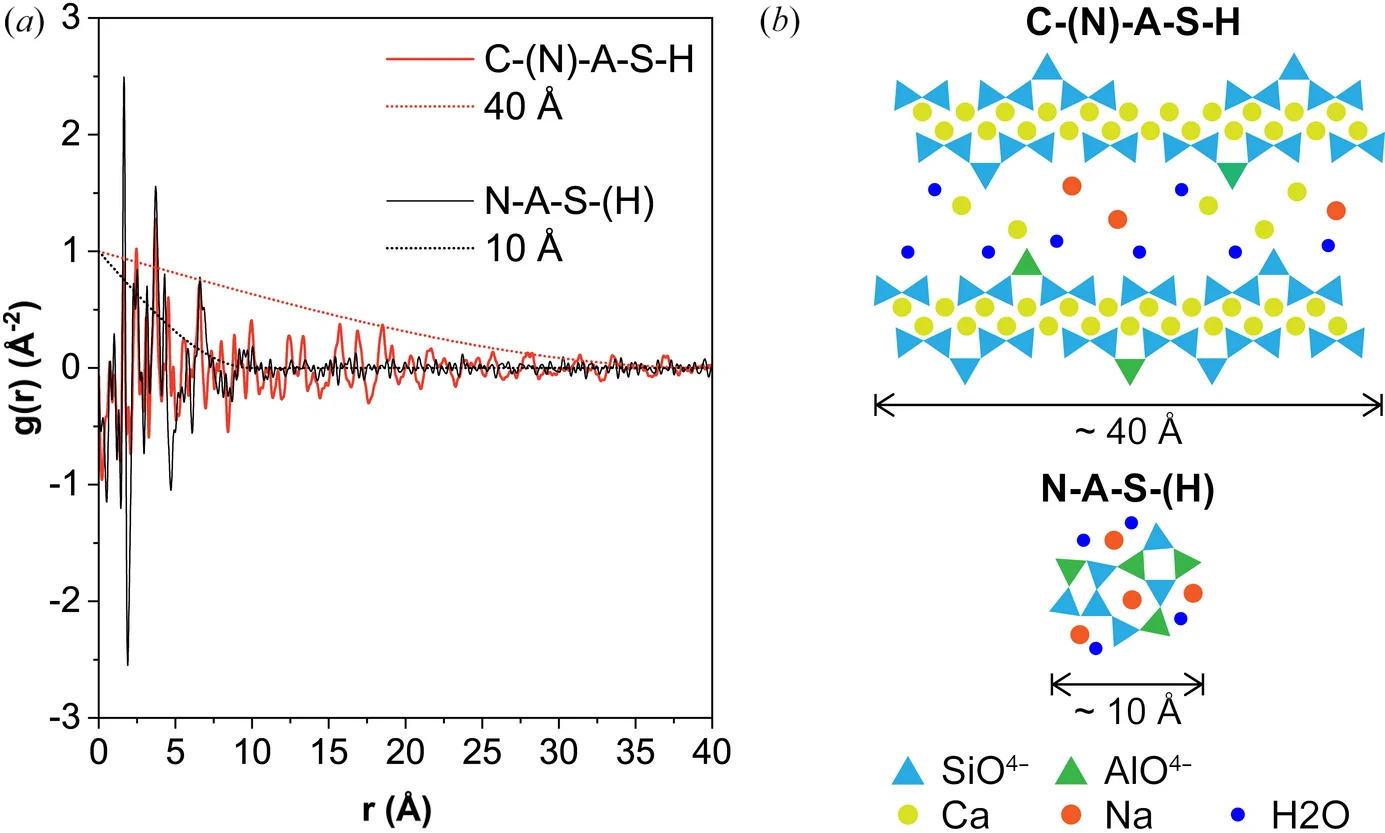
(*a*) Comparison of the PDF curves of C-(N)-A-S-H gel and N-A-S-(H) gel with envelope function (Howell *et al.*, 2006 ▸). (*b*) Schematic illustration of the structures of C-(N)-A-S-H gel and N-A-S-(H) gel.

In order to quantitatively visualize the difference in long-range ordering of C-(N)-A-S-H and N-A-S-(H) gels formed in AAS and AAM systems, PDF curves of two gels were plotted in Fig. 10 ▸(*a*). The envelope functions [*f*_e_(*r*, *d*)] with two different correlation lengths, 10 and 40 Å, were also plotted in the figure as visual guides to compare the *r*-range over which coherent atom-pair correlations persist in the two gel-associated residual PDF signals. The envelope function has been successfully adapted to assess the mean correlation length of nanoparticles as well as cementitious materials in PDF curves (Garg *et al.*, 2019 ▸; Hong *et al.*, 2016 ▸). The envelope function is defined as
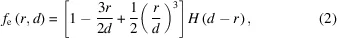
where *r* is the distance between atoms in Å, *d* is the mean particle size in Å, and *H* is the Heaviside step function where *H*(*d* − *r*) goes to zero when *r* > *d* (Howell *et al.*, 2006 ▸). The comparison curves clearly showed the intrinsic difference between the atomic scale ordering of C-(N)-A-S-H and N-A-S-(H) gels. N-A-S-(H) gel formed in the AAM system predominantly showed an amorphous structure which structural features limited to 10 Å, whereas C-(N)-A-S-H gel in the AAS system presented higher long-range order up to 40 Å, as shown in Fig. 10 ▸(*b*). This longer correlation length could be interpreted as greater long-range structural coherence in C-(N)-A-S-H, which may arise from a combination of coherent crystallite size, crystallinity, and network connectivity. This contrast is consistent with previous structural descriptions of these gels, where C-(N)-A-S-H is commonly described as a nano-crystalline C-S-H/tobermorite-like gel, while N-A-S-(H) is described as a highly disordered aluminosilicate framework.

Although this research mainly focuses on the difference between high Ca and low Ca alkali-activated systems, activator chemistry can also influence the development of gel structure. Haha *et al.* (2011 ▸) showed that NaOH and hydrous sodium metasilicate activators produce distinct Al-bearing C-S-H structures in alkali-activated slag, with NaOH activation leading to denser, less water-rich C-S-H. In blended slag–metakaolin systems, the silica modulus of the activator and slag/metakaolin ratio affect reaction kinetics, gel crosslinking, and the formation of C–S–H, Na-substituted C–S–H, and zeolitic aluminosilicate reaction products (Bernal *et al.*, 2011 ▸). In low Ca metakaolin systems, the speciation of soluble silicate in the activator affects the polymerization of the aluminosilicate framework (Poggetto *et al.*, 2024 ▸). These studies suggest that activator chemistry can alter the kinetics and structural development of alkali-activated gels. Therefore, future studies examining the effects of activator type and composition would provide further insight into how the C-(N)-A-S-H and N-A-S-(H) gels evolve under different activation conditions.

The results of this work show the clear difference in the evolution of local atomic structures between two different types of aluminosilicate precursors, blast furnace slag and metakaolin, which represent high calcium and low calcium precursors, respectively. The isothermal calorimetry results (Fig. 2 ▸) showed that the reaction kinetics of two alkali-activated aluminosilicate precursors were significantly different. The reaction kinetics of AAS showed a clear dormant period similar to conventional Portland cement-based systems, whereas the reaction kinetics of AAM did not have such an induction period. In addition, the evolution of the local atomic structure of AAM and AAS via PDF analysis (Figs. 4 ▸ and 5 ▸) was highly dissimilar. Most importantly, as shown in Fig. 10 ▸, there was a significant difference in the long-range ordering of the reaction products of AAS and AAM. In the high calcium system, C-(N)-A-S-H gel continued to grow in the long-range region (∼40 Å) and showed the nano-crystalline structure after ∼5 months of reaction. In contrast, N-A-S-(H) gel in the AAM paste did not show the atomic correlation beyond ∼10 Å even after the long-term reaction had taken place.

## 4. Conclusion

In this study, *in situ*X-ray total scattering analysis and isothermal calorimetry were utilized in tandem to investigate the atomic ordering and reaction kinetics of low Ca and high Ca alkali-activated systems. Ground-granulated blast furnace slag and metakaolin were used as the representation of high and low Ca alkali-activated precursors, respectively. Heat flow data obtained from isothermal calorimetry revealed contrasting reaction behaviors of alkali-activated slag and metakaolin; where alkali-activated slag showed a clear induction period, alkali-activated metakaolin did not have an induction period. In addition, the growth of gel-associated atom–atom correlations in the *in situ* PDF, which are related to the main binding gels in both alkali-activated systems, has excellent agreement with cumulative heat from isothermal calorimetry results (*R*^2^ > 0.97). Specifically, the Ca–T peak in alkali-activated slag, located at ∼3.7 Å, and the T–O* peak in alkali-activated metakaolin centered at ∼4.3 Å can be used to track the C-(N)-A-S-H and N-A-S-(H) gel formation for each system. This result highlights that time-resolved PDF peak evolution can serve as a structural indicator of reaction progress and directly links heat evolution to the formation of the dominant binder gels. Moreover, the isolated PDF results of C-(N)-A-S-H and N-A-S-(H) gels had a significant difference in long-range atomic ordering above ∼10 Å. While N-A-S-(H) gel did not show atomic correlations above ∼10 Å, C-(N)-A-S-H gel evolved to the nano-crystalline region (∼40 Å) with the reaction progress, suggesting an inherent difference in the atomic ordering of high and low Ca alkali-activated systems.

## Supplementary Material

Fig. S1: X-ray pair distribution function for alkali-activated slag and alkali-activated metakaolin. DOI: 10.1107/S1600577526006776/vl5057sup1.pdf

## Figures and Tables

**Figure 9:**
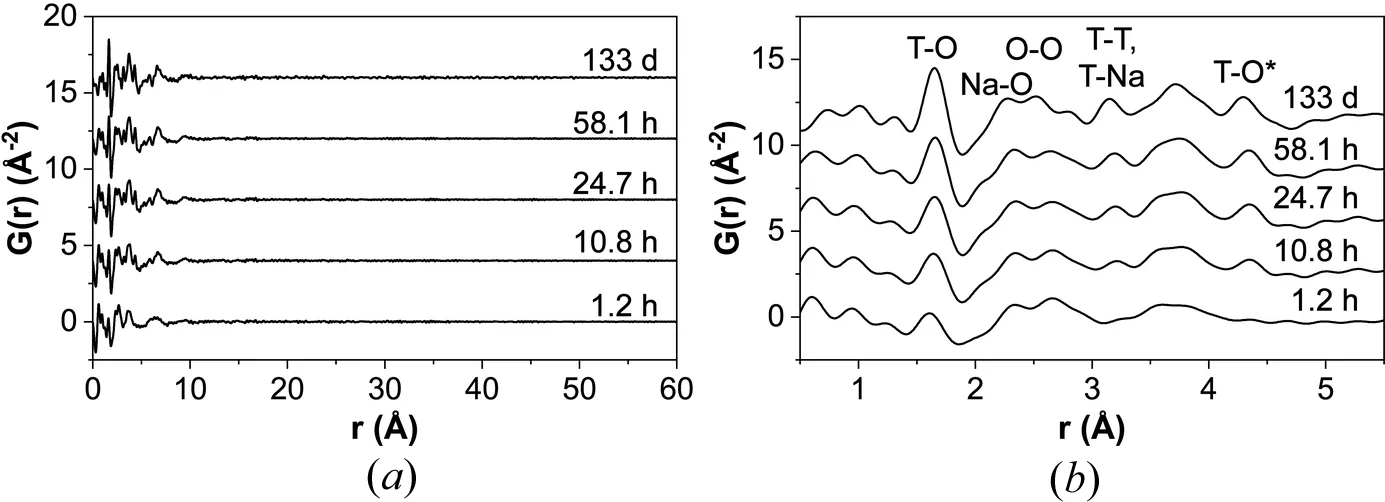
Pair distribution function curves of N-A-S-(H) gel in the AAM paste over (*a*) long *r* range (0–60 Å) and (*b*) short *r* range (0.5–5.5 Å). The curves were obtained by subtracting the unreacted metakaolin from the original pair distribution patterns at the specific reaction time.
