# Supplementary material for: Time-resolved synchrotron X-ray total scattering of alkali-activated materials with high and low calcium contents
Source: J Synchrotron Radiat. 2026 Aug 3;33(Pt 5):1406–17. doi: 10.1107/S1600577526006776 (PMC13549352; doi:10.1107/S1600577526006776)
Supplement: Supplementary file 1 [file s-33-01406-sup1.pdf]

JOURNAL OF SYNCHROTRON RADIATION

Volume 33 (2026)

Supporting information for article:

Time-resolved Synchrotron X-ray Total Scattering of Alkali-activated Materials with High and Low Calcium Contents

Hyeonseok Jee and Nishant Garg

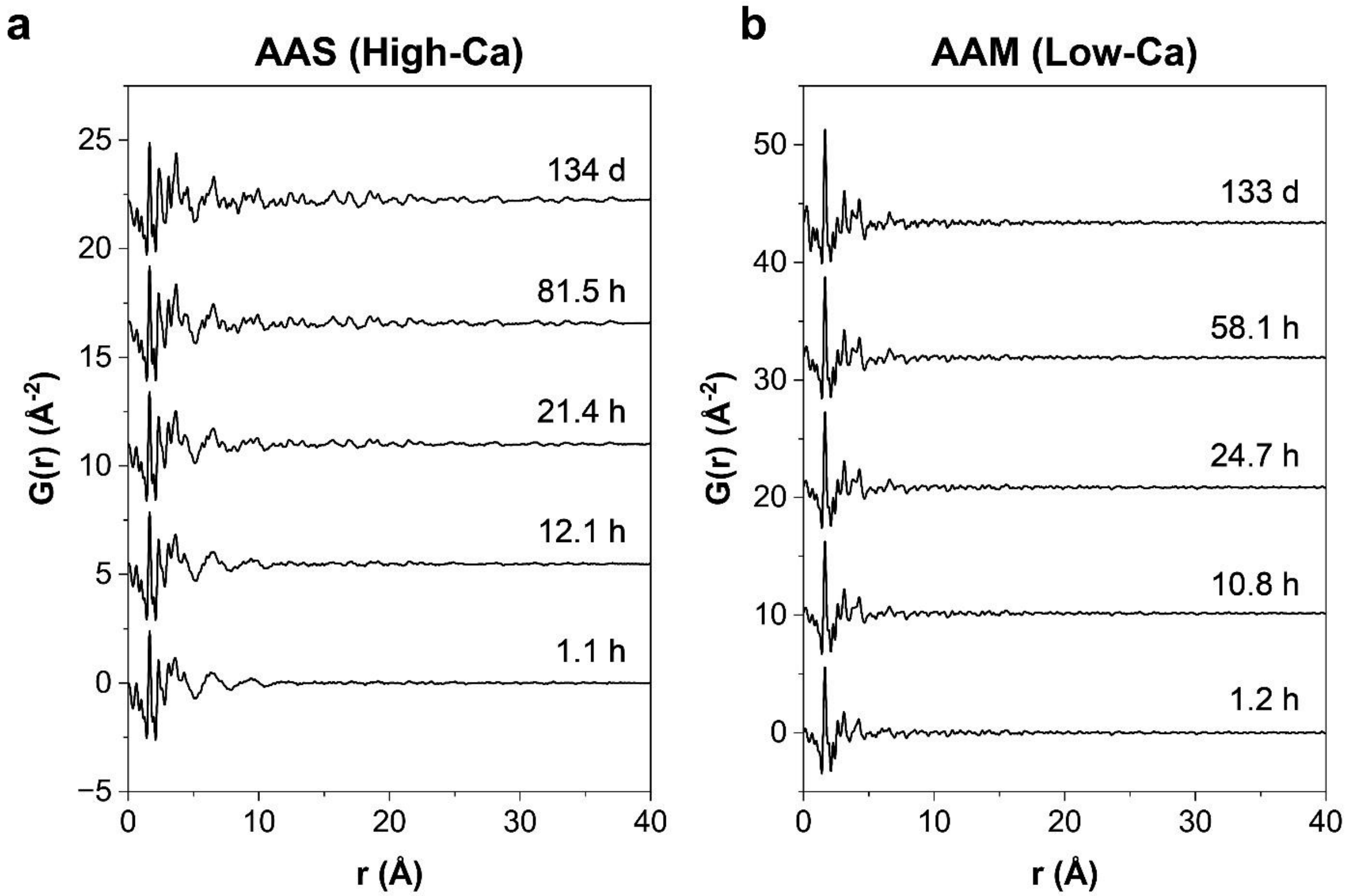

Figure S1 X-ray pair distribution function for the (a) alkali-activated slag and (b) alkali-activated metakaolin.
